# Prognostic models for upper urinary tract urothelial carcinoma patients after radical nephroureterectomy based on a novel systemic immune-inflammation score with machine learning

**DOI:** 10.1186/s12885-023-11058-z

**Published:** 2023-06-22

**Authors:** Jianyong Liu, Pengjie Wu, Shicong Lai, Jianye Wang, Huimin Hou, Yaoguang Zhang

**Affiliations:** 1 Department of Urology, Beijing Hospital, National Center of Gerontology, Institute of the Geriatric Medicine, Chinese Academy of Medical Sciences, No. 1 DaHua Road, Dong Dan, Beijing, China; 2 Graduate School of Peking Union Medical College, Chinese Academy of Medical Sciences, Beijing, China; 3 Beijing Hospital Continence Center, Beijing, China; 4 Department of Urology, Peking University People’s Hospital, 100044 Beijing, China

**Keywords:** Upper urinary tract urothelial carcinoma, Systemic immune-inflammation score, Random survival forest, Prognosis, Risk stratification

## Abstract

**Purpose:**

This study aimed to evaluate the clinical significance of a novel systemic immune-inflammation score (SIIS) to predict oncological outcomes in upper urinary tract urothelial carcinoma(UTUC) after radical nephroureterectomy(RNU).

**Method:**

The clinical data of 483 patients with nonmetastatic UTUC underwent surgery in our center were analyzed. **Five** inflammation-related biomarkers were screened in the Lasso-Cox model and then aggregated to generate the SIIS based on the regression coefficients. Overall survival (OS) was assessed using Kaplan-Meier analyses. The Cox proportional hazards regression and random survival forest model were adopted to build the prognostic model. Then we established an effective nomogram for UTUC after RNU based on SIIS. The discrimination and calibration of the nomogram were evaluated using the concordance index (C-index), area under the time-dependent receiver operating characteristic curve (time-dependent AUC), and calibration curves. Decision curve analysis (DCA) was used to assess the net benefits of the nomogram at different threshold probabilities.

**Result:**

According to the median value SIIS computed by the lasso Cox model, the high-risk group had worse OS (*p*<0.0001) than low risk-group. Variables with a minimum depth greater than the depth threshold or negative variable importance were excluded, and the remaining six variables were included in the model. The area under the ROC curve (AUROC) of the Cox and random survival forest models were 0.801 and 0.872 for OS at five years, respectively. Multivariate Cox analysis showed that elevated SIIS was significantly associated with poorer OS (*p*<0.001). In terms of predicting overall survival, a nomogram that considered the SIIS and clinical prognostic factors performed better than the AJCC staging.

**Conclusion:**

The pretreatment levels of SIIS were an independent predictor of prognosis in upper urinary tract urothelial carcinoma after RNU. Therefore, incorporating SIIS into currently available clinical parameters helps predict the long-term survival of UTUC.

## Background

Upper urinary tract urothelial cell carcinomas (UTUCs) is located in the upper (pyelocaliceal cavities and ureter) urinary tract, and pyelocaliceal tumors are approximately twice as common as ureteral tumors [1]. UTUCs are relatively rare types of urological disease, representing roughly 5–10% of all urothelial tumors [1]. The prognosis is usually poor because it quickly appears to have a propensity for local relapse, intravesical recurrence, and distant metastasis. The previous literature has shown that the 5-year disease-free survival (DFS) rate was 50% for patients with UTUC with local muscular invasion, and for those with advanced disease, the rate declined to 10% [2]. Moreover, the rate of intravesical recurrence (IVR) differs significantly according to the literature, and data range from 20 to 69% [3–7]. Radical nephroureterectomy (RNU) with bladder cuff excision is considered the gold standard treatment of nonmetastatic, high-grade UTUC because there is a considerable risk of tumor recurrence in the distal ureter and its orifice. There has been an improvement in current treatment modalities, such as chemotherapy, surgery, and immunotherapy, but the overall outcome of patients with UTUC remains dismal.

Cancer-associated inflammation is considered the seventh hallmark of cancer [8]. Amounting evidence has suggested that systemic inflammatory response plays a critical role in the development and progression of tumors [9, 10]. In the tumor microenvironment, inflammation promotes the proliferation and survival of tumor-initiating cells, angiogenesis, metastasis, dysregulation of specific immunity, hormone resistance, and decreased response to chemotherapeutic drugs [8]. Elevated systemic inflammatory responses may be an important indicator of cancer progression and prognosis [11]. Several preoperative peripheral blood biomarkers may be markers for predicting the patient’s prognosis due to their correlation with baseline inflammatory and immune status [12]. For clinicians, it is very basic and important to detect the peripheral blood of patients before surgery. Systemic inflammation can be assessed by various markers measured in routine blood tests or by new indicators derived from these markers through basic mathematical operations. It has been reported that preoperative serum inflammation biomarkers, including the neutrophil-to-lymphocyte ratio (NLR)[13], the platelet-to-lymphocyte ratio (PLR) [14], the monocyte-to-lymphocyte ratio (MLR) [15, 16], and the systemic immune-inflammation index (SII: neutrophils*platelets/lymphocytes) [17], have a high prognostic impact and could serve as biomarkers of cancer incidence risk in many types of tumors. In addition, several studies have revealed that the systemic inflammation response index (SIRI), based on neutrophil, monocyte, and lymphocyte count, is an independent prognostic factor for different cancers [18–22]. Previous studies have demonstrated that several systemic inflammatory markers worsen OS in patients with UTUC[23–25]. Without exception, these indicators are generated using two or three blood characteristics through basic mathematical operations such as addition and division. It is unclear which combination of blood features is a strong marker of prognosis in UTUC patients. Some scholars have previously developed new prognostic markers through the combination of various inflammatory indicators, which can more accurately predict the survival of cancer patients [26–28]. Therefore, we also believe that combining these markers can predict clinical survival more accurately than using a single marker.

Recently, the rapid development of machine learning has made it increasingly used in medical research, where it can process many input features and generate accurate prediction models. For example, random Survival Forest (RSF) is a random forest method for analyzing right-censored survival data. It introduces new survival splitting rules for growing survival trees and a new missing data algorithm for imputing missing data [29]. Because RSF can construct multiple decision trees to predict the outcome and simulate the nonlinear effects and complex interactions among factors. Thus, a higher level of accuracy is achieved. In addition, researchers have recently applied machine learning to the prediction and prognosis of cancer [30–32].

Hence,this study aimed to develop a new and powerful prognostic indicator for UTUC through the combination of systemic inflammation markers in blood. Furthermore, based on machine learning, we planned to explore the prognostic value of the novel systemic immune-inflammation score (SIIS) for prognosis in UTUC patients treated with RNU and aimed to provide appropriate and individualized therapy in clinical treatment.

## Patients and methods

### Patients

We retrospectively analyzed the clinicopathological data of patients with UTUC who underwent RNU with bladder cuff excision at our institution between March 1996 and June 2021. Patients with clinical evidence of infection, such as fever (>38 °C) or chronic inflammatory diseases, were excluded from the study. Patients with a history of previous/concomitant bladder cancer, receiving neoadjuvant chemotherapy, preoperative radiotherapy, or both were excluded from enrolment. The study did not include patients with missing SIIS data (including neutrophil, monocyte, platelet, and lymphocyte counts). Patients with evidence of metastatic disease at the time of surgery and those who did not undergo RNU were also excluded.

### Clinical and pathologic characteristics

Collected clinical and pathological parameters included agender, age, symptoms, type of operation preoperative, NLR, MLR, PLR, SII, SIRI, tumor location, tumor side, tumor size, presence of preoperative hydronephrosis and hematuria, pT and pN stage, grade, lympho-vascular invasion (LVI), multifocality, the presence of concomitant CIS, and surgical margin status.

Preoperative computed tomography (CTU), magnetic resonance imaging (MRI), or intravenous pyelography were used to measure the tumor side and location. Tumor size was classified into two groups (≤ 5 cm or >5 cm). Tumor location was divided into PUJ (pelvi-ureteric junction), ureter, and both. Multifocality was defined as the simultaneous presence of tumors at discontinuous locations or two or more tumors. All patients underwent RNU with open or laparoscopic surgical excision of the bladder cuff. In addition, a preoperative imaging study before surgery performed lymph node dissection in patients with suspiciously enlarged lymph nodes.

For grading, the 1998 World Health Organization/International Society of Urologic Pathology consensus classification was used, and staging was assessed according to the American Joint Committee on Cancer TNM Classification, 7th edition. SIRI was calculated using the following equation: SIRI = neutrophil × monocyte/lymphocyte. All patients’ complete blood count (CBC) samples were collected within one month before the surgery. Adjuvant chemotherapy or intravesical instillation was not routinely proposed for all patients but up to the patient’s tumor stage and grade.

### Follow-up

Patients were observed every 3–4 months in the first year after surgery, every 6 months from the second year through the third year, and every 12 months after that. The follow-up consisted of cystoscopy, routine blood testing, urinary cytology, and chest and abdominal radiography. The primary endpoints were overall survival (OS) (as the date of surgery to the date of death from any cause).

### Features extraction

A total of clinical and histopathological parameters were collected. Random survival forest can rank variable importance (VIMP). VIMP and minimum depth method are the most commonly used methods: a variable VIMP value less than 0 indicates that the variable reduces the accuracy of the prediction, while a VIMP value greater than 0 indicates that the variable improves the accuracy of prediction; the minimum depth method gives the importance of each variable to the outcome event by calculating the minimum depth when running to the final node. We performed variable screening by combining the two methods performed by the randomForestSRC R package. Besides, we used the variance inflation factor (VIF) method to find the features causing the multicollinearity and then removed them (VIF ≥ 10).

### Statistical analysis

Statistical analysis was performed with the use of R4.2.1. Surv_cutpoint function in R package survminer was used to calculate the optimal cutoff value of NLR, MLR, PLR, SII, and SIRI. If the level of each serum marker was higher than or equal to the optimal cutoff value, the score was 1; otherwise, the score was 0. Five serum markers mentioned above were retained by application of the LASSO-Cox regression model with a minimum of λ. Then, the regression coefficient of each tumor marker was calculated with the optimal λ value, and we used Pearson correlation analysis to evaluate the correlation between inflammatory indicators. A significant correlation was considered when the coefficient |R| > 0.4 and *p* < 0.05. Finally, the correlated indicators were removed and the SIIS was calculated according to the serum marker level and its related regression coefficient. Finally, the median score of all individuals was taken as the risk cutoff value, and all UTUC individuals were divided into high-risk or low-risk groups. The Chi-square test compared the relationships between SIIS and other clinicopathological parameters. Survival patterns were identified using the means of the Kaplan-Meier curves, and the log-rank test compared significant differences. Additional subgroup analyses were completed according to agender, LVI, pathological T stage, and tumor grade. Based on the features extraction method, a random survival forest model was established by the rfsrc function in the randomForestSRC package. The Harrell’s Concordance Index was used to evaluate the discrimination of the predictive model: C-index = 1-Error rate. Multivariable analyses using the Cox proportional hazards model were performed to identify risk factors for OS after RNU. Meanwhile, a prognostic nomogram was constructed based on the above clinicopathological factors, providing optimum accuracy in predicting OS. The receiver operating characteristic curves (ROC), the calibration curve, and decision curve analysis (DCA) evaluated the nomogram’s discrimination, calibration, and clinical usefulness. A value of *p*<0.05 was considered to be statistically significant.

## Result

### The optimal cutoff values determined by R package survminer and their prognostic role

**Fig. 1:**
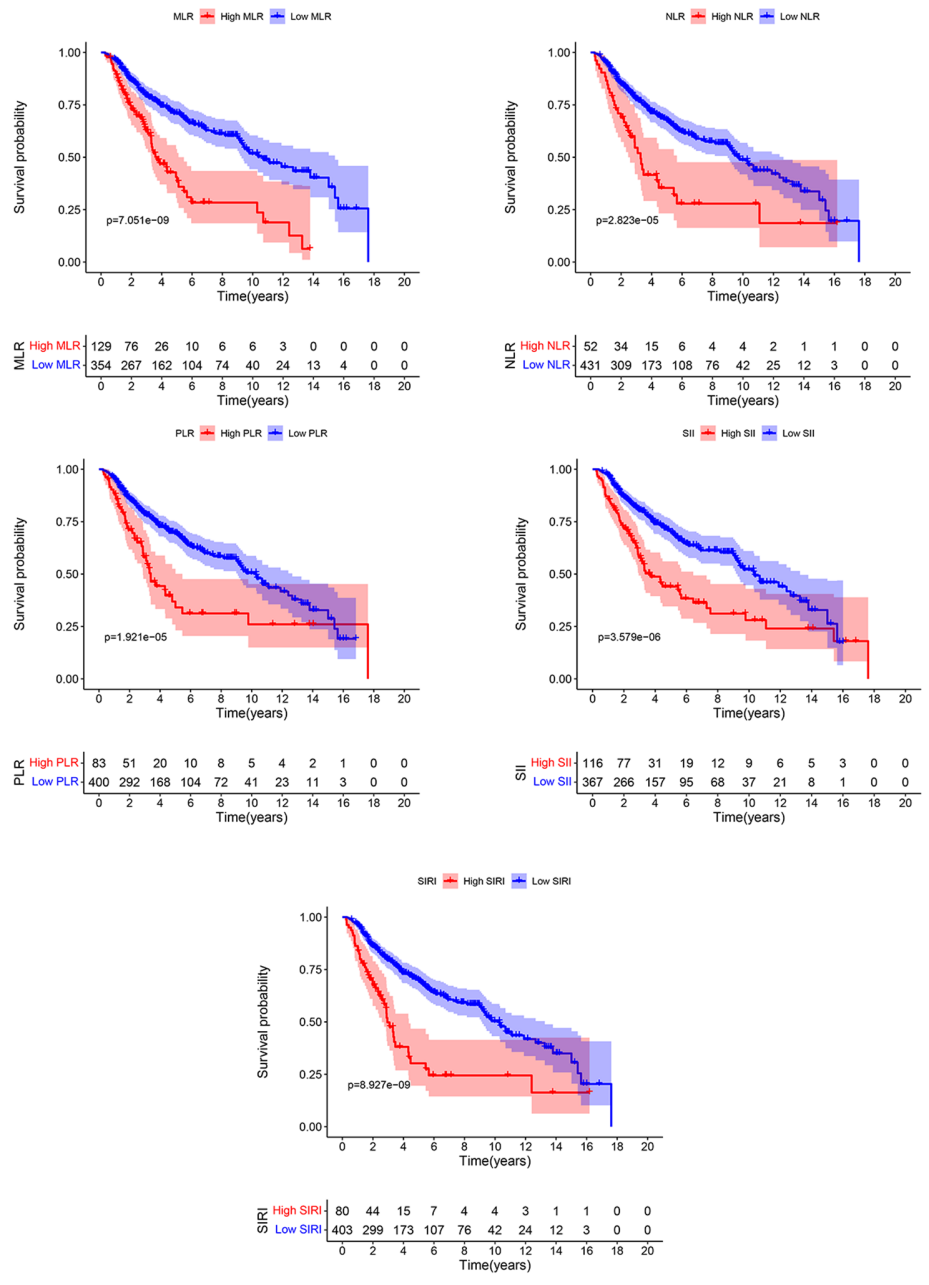
The Kaplan-Meier survival curve for the five serum markers based on the optimal cut-off point

Using the surv_cutpoint function of the survminer package, the optimal cutoff point was determined for continuous variables, as shown in Table 1. The optimal cutoff values of NLR, MLR, PLR, SII, and SIRI concerning overall survival were 3.985, 0.305, 180.233, 710.608, and 1.706, respectively. The Kaplan*-*Meier survival curve for the five serum markers is shown in Fig. 1. The survival curves for preoperative NLR, MLR, PLR, SII, and SIRI levels showed shorter OS times in patients with higher levels than those with lower serum makers. Thus, these serum markers were incorporated into the subsequent analysis.

**Table 1:** The optimal prognostic cutoff value of each Serum marker

Serum marker	Cutoff	Group	No. of patients N (%)
NLR	3.985	<3.985	431 (89.2%)
		≥3.985	52 (10.8%)
MLR	0.305	<0.305	354 (73.3%)
		≥0.305	129 (26.7%)
PLR	180.233	<180.233	400 (82.8%)
		≥180.233	83 (17.2%)
SII	710.608	<710.608	367 (76.0%)
		≥710.608	116 (24.0%)
SIRI	1.706	<1.706	403 (83.4%)
		≥1.706	80 (16.6%)

**Abbreviations: NLR, neutrophil-to-lymphocyte ratio; MLR, the monocyte-to-lymphocyte ratio; PLR, platelet-to-lymphocyte ratio; SII, systemic immune-inflammation index; SIRI, systemic inflammation response index**

### Calculation of systemic Immune-Inflammation score (SIIS)

**Fig. 2:**
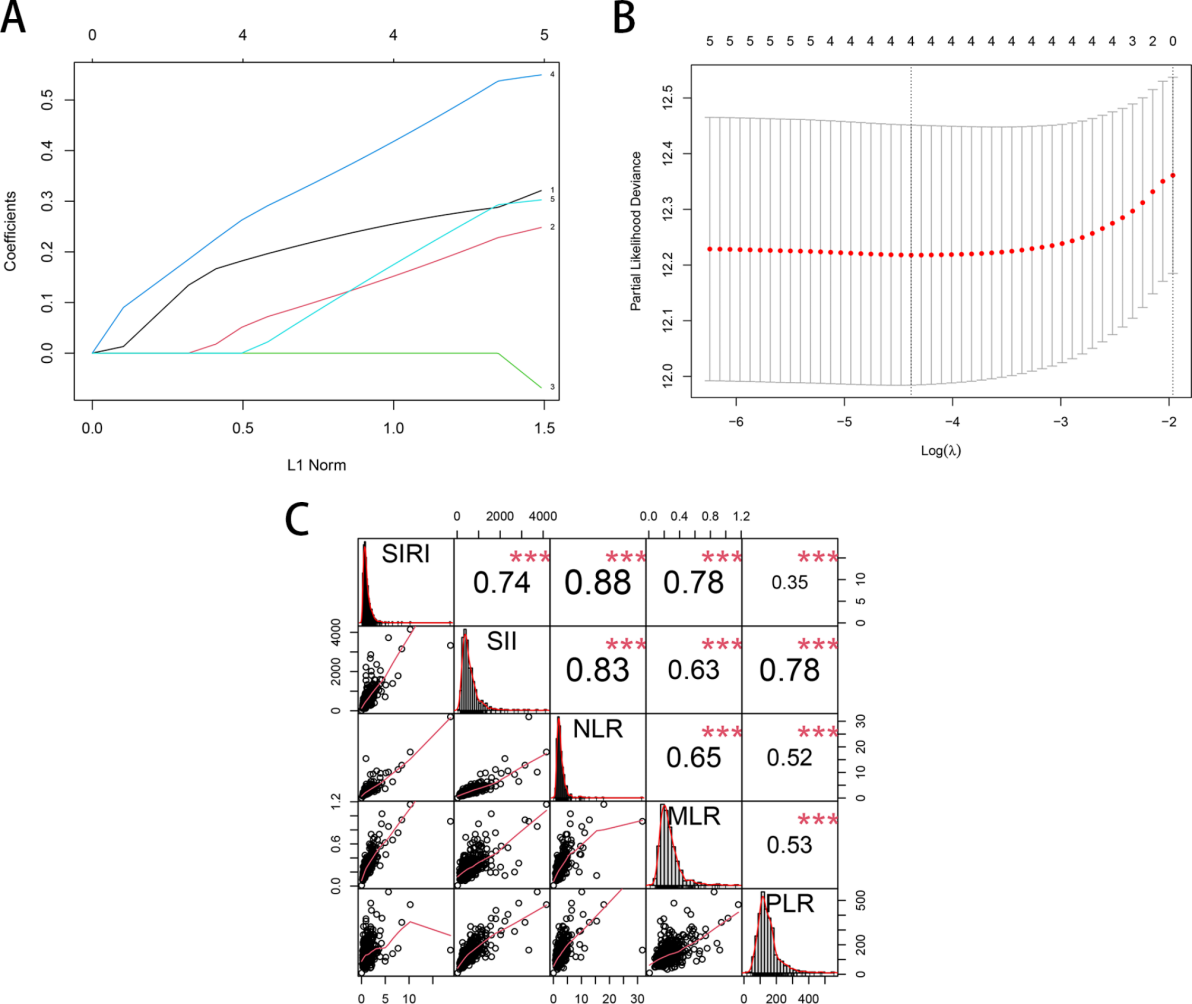
The Construction of the systemic immune-inflammation score (SIIS). **(A)** LASSO coefficient profiles of the 5 serum markers. **(B)** A coefficient profile plot was produced against the log (lambda) sequence in the LASSO model. **(C)** The Pearson correlation analysis among SIRI, SII, MLR and PLR

**Fig. 3:**
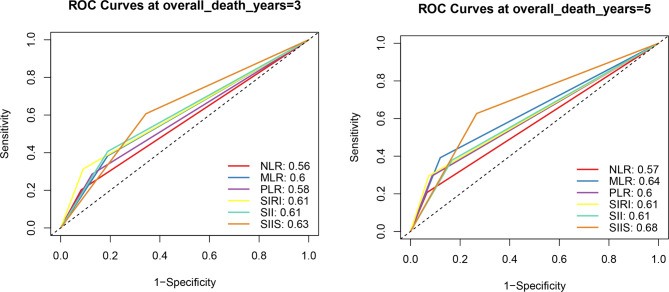
Receiver operator characteristic (ROC) analysis of six markers for predicting overall-survival at three years (**A**) and five years (**B**)

The continuous variables were transformed into dichotomous ones according to the above-cut-off value. Thus, the serum marker levels of patients could be divided into two subgroups: those with a serum marker level above or equal to the cutoff value (1 score) and those with a serum marker level below it (0 scores). Next, the five serum markers were subjected to the least absolute shrinkage and selection operator (LASSO) Cox regression analysis. The calculation of the regression coefficient is visualized in Fig. 2A. When four variables were included, the model achieved the best performance (Fig. 2B). The regression coefficient of NLR turned to zero, while the remaining serum markers were included in the simplified lasso Cox model (Table 2). The Pearson coefficient analysis showed that SIRI and PLR were not significantly correlated (Fig. 2C). Then, each patient’s systemic immune-inflammation score (SIIS) was calculated based on the score levels of the two serum markers and the coefficients from the LASSO Cox regression analysis. SIIS = SIRI*0.2835417 + PLR*0.2739229. Finally, the patients were divided into low-risk and high-risk groups according to the median value of the SIIS. The time-dependent ROC curve also indicated that the AUC of the SIIS was higher at three and five years compared with other markers (Fig. 3).

**Table 2:** Regression coefficients of the lasso Cox model

Serum marker	*β* ^*^
NLR	0
MLR	0.5166279
PLR	0.2739229
SII	0.2148603
SIRI	0.2835417

**Abbreviations: NLR, neutrophil-to-lymphocyte ratio; MLR, the monocyte-to-lymphocyte ratio; PLR, platelet-to-lymphocyte ratio; SII, systemic immune-inflammation index; SIRI, systemic inflammation response index**

**Fig. 4:**
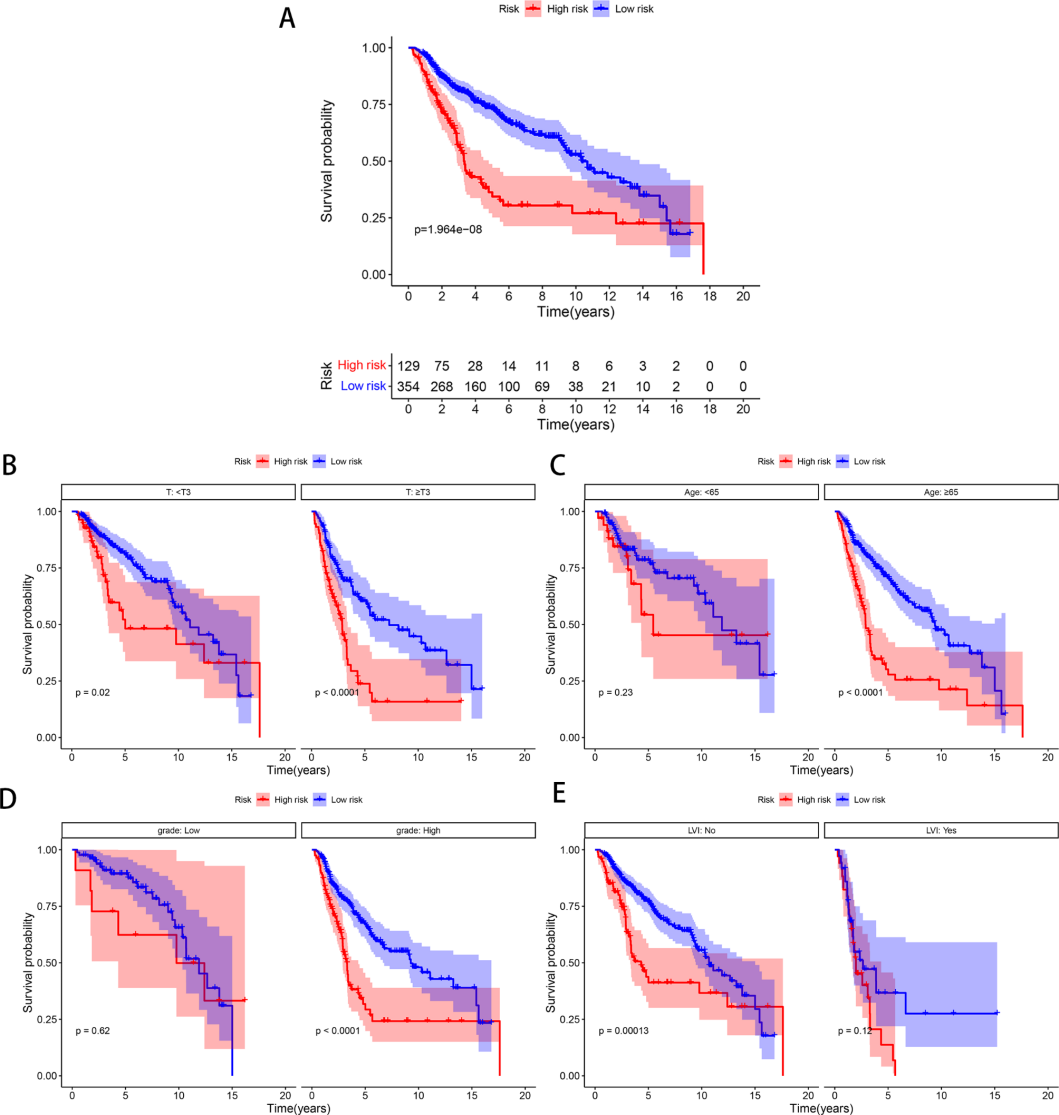
Relationship between SIIS and Clinical Features. **(A)** K-M analyses of OS between high- and low-risk groups. Subgroup analysis based on T stage **(B)**, age **(C)**, tumor grade **(D)**, and LVI **(E)**, Kaplan–Meier curves of OS which was stratified according to SIIS for UTUC patients receiving RNU

### Patient characteristics

The clinical and pathological characteristics of the patients with UTUC are detailed in Table 3. Among the 483 patients, there were 354 low-risk and 129 high-risk patients (low-risk to high-risk ratio 2.74:1). Overall, 14.5% of patients had LVI, and 72.7% of cases had positive urine pathology. The two groups significantly differed in gender, LVI, pathological T stage, pN, and tumor grade. In addition, patients with high risk had lower OS than those with low risk (39.8 versus 57.8 months, *p* < 0.001).

**Table 3:** Baseline and Clinicopathological Characteristics of UTUC Patients

	High risk	Low risk	*p*-value
	*N = 129*	* N = 354*	
Sex			0.001
Female	50 (38.8%)	197 (55.6%)	
Male	79 (61.2%)	157 (44.4%)	
Age (years)			0.216
<65	34 (26.4%)	116 (32.8%)	
≥65	95 (73.6%)	238 (67.2%)	
BMI (kg/m^2)^			0.848
<25	77 (59.7%)	206 (58.2%)	
≥25	52 (40.3%)	148 (41.8%)	
Side			0.617
Left	70 (54.3%)	203 (57.3%)	
Right	59 (45.7%)	151 (42.7%)	
Site			0.514
Pelvis	53 (41.1%)	153 (43.2%)	
Ureter	65 (50.4%)	181 (51.1%)	
Both	11 (8.53%)	20 (5.65%)	
Approach			0.689
Laparoscopic	78 (60.5%)	205 (57.9%)	
Open	51 (39.5%)	149 (42.1%)	
Ureteroscopy			0.918
No	103 (79.8%)	286 (80.8%)	
Yes	26 (20.2%)	68 (19.2%)	
Urine pathology			0.327
Negative	40 (31.0%)	92 (26.0%)	
Positive	89 (69.0%)	262 (74.0%)	
Hydronephrosis			0.341
No	25 (19.4%)	85 (24.0%)	
Yes	104 (80.6%)	269 (76.0%)	
Multifocality			0.121
No	106 (82.2%)	312 (88.1%)	
Yes	23 (17.8%)	42 (11.9%)	
Size (cm)			0.171
<5	106 (82.2%)	310 (87.6%)	
≥5	23 (17.8%)	44 (12.4%)	
LVI			<0.001
No	95 (73.6%)	318 (89.8%)	
Yes	34 (26.4%)	36 (10.2%)	
Tis			0.756
No	124 (96.1%)	336 (94.9%)	
Yes	5 (3.88%)	18 (5.08%)	
T stage			<0.001
T1	18 (14.0%)	110 (31.1%)	
T2	38 (29.5%)	105 (29.7%)	
T3	62 (48.1%)	128 (36.2%)	
T4	11 (8.53%)	11 (3.11%)	
Margin			0.119
Negative	123 (95.3%)	347 (98.0%)	
Positive	6 (4.65%)	7 (1.98%)	
pN			0.020
N0&Nx	114 (88.4%)	336 (94.9%)	
N+	15 (11.6%)	18 (5.08%)	
Grade			<0.001
Low	11 (8.53%)	83 (23.4%)	
High	118 (91.5%)	271 (76.6%)	

**Abbreviations: BMI, body mass index; CIS, carcinoma in situ; LVI, lympho-vascular invasion**

### Relationship between SIIS and Clinical features

Kaplan-Meier analysis revealed that patients with high risk had a significantly poorer OS compared with patients with low risk (*p* < 0.001) (Fig. 4A). In subgroup analyses, the K-M survival curves revealed a higher survival probability for the low-risk group both in the T<3 (*p* = 0.020) and T ≥ 3 (*p* < 0.001) (Fig. 4B). In the age ≥ 65(*p* < 0.001) and high-grade group(*p* < 0.001), the high-risk group had a worse prognosis, while there was no significant difference in the age<65and low-grade group between the two groups (Fig. 4C and D). By subgroup analysis, high-risk predicted decreased OS (*p* < 0.001) in patients without LVI, which was not observed in patients with LVI (Fig. 4E).

**Fig. 5:**
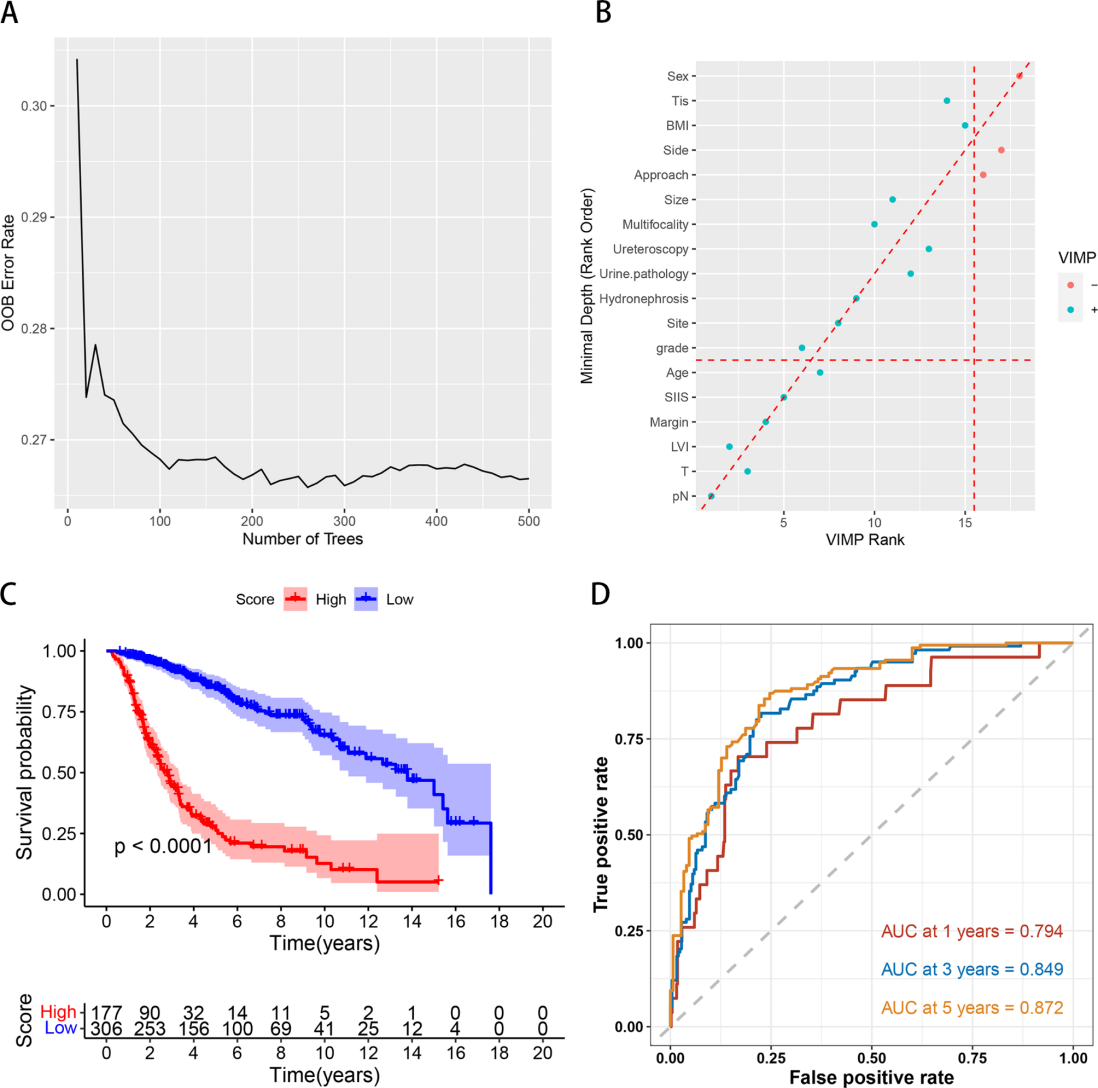
Random survival forest. **(A)** The prediction error rate for random survival forests of 500 trees. **(B)** Comparing minimal depth and variable importance (VIMP) rankings. **(C)** K-M analyses of OS based on RSF-score. **(D)** Time-dependent ROC curves of the RSF model

**Fig. 6:**
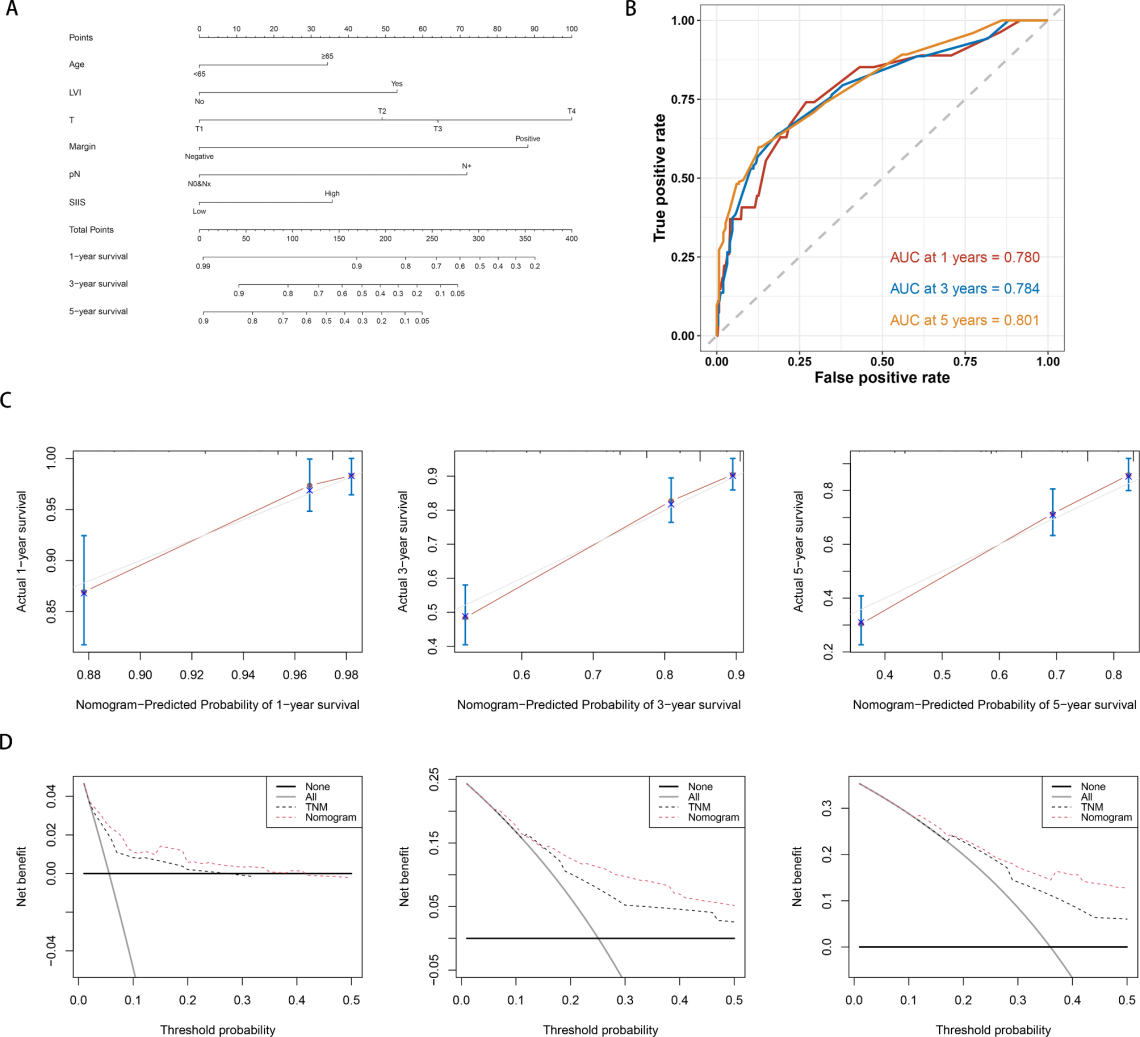
Establishment of SIIS-related clinicopathologic nomogram. **(A)** Development of a prognostic nomogram to predict 1-, 3-, and 5-year OS in UTUC patients. **(B)** Time-dependent ROC curves of the nomogram. **(C)** The calibration curves for predicting 1-, 3- and 5-year OS. **(D)** Decision curve analysis (DCA) to assess the clinical decision-making benefits of the nomogram

**Fig. 7:**
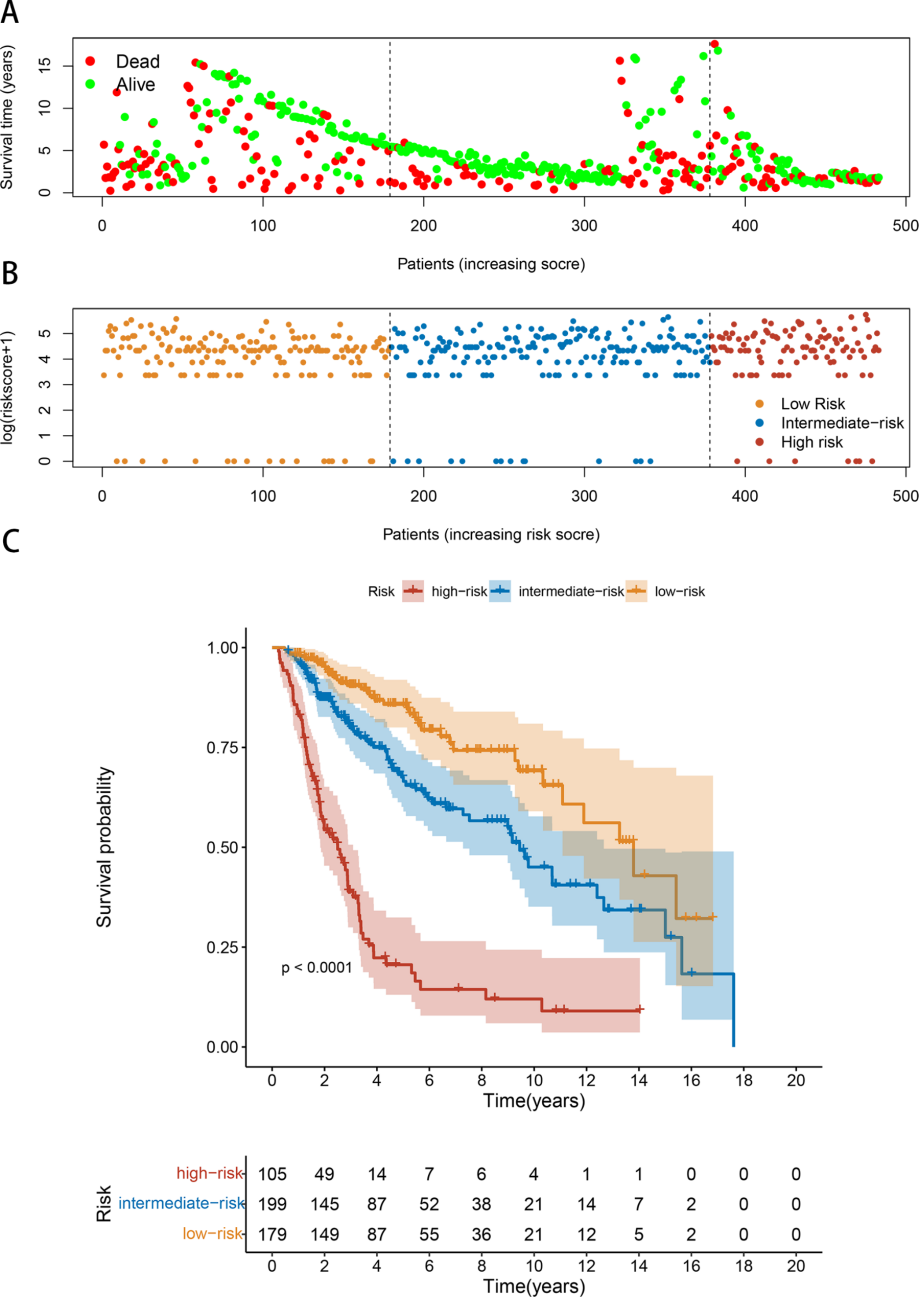
Risk stratification for UTUC patients. **(A)** Risk heatmap. **(B)** Survival status map. Patients’ **(C)** Survival curves showed the OS of the high-risk, intermediate-risk, and low-risk groups

### Random survival forest model and Cox regression model

The R software RandomForestSRC package was used to construct the RSF. The model generates 500 binary survival trees, and it can be seen from Fig. 5A that when the number of survival trees was increased to a certain number, the out-of-bag error (OOB error) rate curve tended to be smooth (0.266), indicating that the number of trees was appropriate. In this paper, 18 variables were processed by the randomForestSRC R package for feature selection. According to the VIMP and minimum depth methods, in Fig. 5B, the blue dot represents a VIMP value greater than 0, and the red represents a VIMP value less than 0. The point higher than the diagonal dotted line represents its VIMP ranking, and the point lower than the diagonal dotted line represents its minimum depth ranking. Finally, in the RSF model built with six variables (including age, SIIS, surgical margin, LVI, T stage, and pN), the most important factor was pN (minimal depth 2.088). Subsequently, the RSF-scores were calculated based on RSF. A cutoff value of 52.983 was calculated using the function “surv_cutpoint” in the R package “survminer”. We divided patients into high- and low-risk groups based on the cutoff value. The Kaplan-Meier survival analysis showed that patients in the high-risk group had significantly higher mortality risk than those in the low-risk group (Fig. 5C, *p* < 0.001). Predictive performance was evaluated using a time-dependent ROC curve. The 1-, 3-, and 5-year AUC values were 0.794, 0.849, and 0.872, respectively (Fig. 5D).

In the univariate Cox regression analysis, all sixvariables that were included in the RSF model remained significant. The result from the univariate analysis showed that age, LVI, T stage, surgical margin, pN, and SIIS (All *p*<0.05) as predictors for OS (Table 4). The SIIS (HR = 1.835, 95%CI: 1.341–2.510, *p*<0.001) was also confirmed as an independent risk factor for OS in multivariate Cox regression analysis (Table 4).

**Table 4:** Univariate and Multivariate Cox Analyses for OS of UTUC Patients

Variable	Univariate Cox analysis			Multivariate Cox analysis		
	HR	95%CI	p-value	HR	95%CI	p-value
Age						
<65	Reference			Reference		
≥65	1.681	1.189–2.376	0.003	1.550	1.088–2.208	0.015
LVI						
No	Reference			Reference		
Yes	3.766	2.668–5.316	<0.001	1.979	1.311–2.987	0.001
T stage						
T1	Reference			Reference		
T2	2.507	1.551–4.052	<0.001	2.086	1.282–3.395	0.003
T3	3.404	2.155–5.376	<0.001	2.511	1.558–4.045	<0.001
T4	12.446	6.821–22.709	<0.001	4.821	2.410–9.643	<0.001
Margin						
Negative	Reference			Reference		
Positive	5.062	2.648–9.678	<0.001	3.285	1.643–6.569	<0.001
N stage						
pN0&Nx	Reference			Reference		
pN+	5.344	3.583–7.972	<0.001	2.668	1.698–4.191	<0.001
SIIS						
Low risk	Reference			Reference		
High risk	2.337	1.723–3.169	<0.001	1.835	1.341–2.510	<0.001

**Abbreviations: LVI, lympho-vascular invasion; SIIS, Systemic Immune-Inflammation Score**

### Development of a Novel Prognostic Nomogram

Sixfactors were included in the nomogram predicting the OS probability of UTUC patients based on multivariate analysis results (Fig. 6A). The ROC curves demonstrated the good discriminative abilities of the nomograms (Fig. 6B). In the prediction of 1-, 3-, and 5-year OS, the AUCs of the nomogram were 0.780, 0.784, and 0.801, respectively. The calibration plots presented excellent agreement between the predicted and observed survival probabilities (Fig. 6C). Additionally, DCA showed that the nomogram had better clinical utilization than the AJCC TNM staging system at different time points (death at 1, 3, and 5 years) (Fig. 6D).

The patients were divided into three groups (low-risk (scores<75), intermediate-risk (75 ≤ scores<125), and high-risk (scores ≥ 125)) according to the score of each factor in the nomogram. TNM stages I, II, III, and IV each represented 0,47, 58, and 100 scores. Age ≥ 65, the presence of LVI, and positive surgical margin represented 28, 43, and 76 scores, respectively. A score of 63and 48 were assigned to individuals with lymph node-positive high SIIS. Scatterplots illustrate the relationship between the survival data and scores (Fig. 7A). The risk score distribution is shown in Fig. 7B. Each group had a different prognosis in the Kaplan-Meier curves for OS (Fig. 7C). Collectively, this nomogram could well stratify patients who are at high risk and guide patient management.

## Discussion

Despite undergoing surgery, patients with UTUC had a poor prognosis and a high recurrence rate. Some factors derived from postoperative data, including tumor stage, tumor grade, tumor necrosis, surgical margins, lymph node involvement, and lympho-vascular invasion (LVI), have been associated with oncological outcomes [1]. Although some preoperative biomarkers, such as NLR, SIRI, PLR, SII, and MLR, are considered independent prognostic factors in patients with UTUC [18, 23, 24, 33, 34], it is not enough to guide clinical decision-making. And as many researchers combine various indicators based on new statistical methods to develop a new prognostic index, it can more accurately predict the prognosis of cancer patients than a single indicator. Therefore, it is very important to develop a new and powerful prognostic indicator for further risk stratification and individualized treatment.

Our study first developed a novel systemic immune-inflammation score (SIIS) based on the Cox regression model and machine learning. Considering collinearity and correlation between different variables and indicators, we utilized dimensionality reduction by the LASSO regression and the Pearson correlation analysis to ease the interference between variables. In addition, numerous studies have incorporated machine learning into clinical practice, such as individualized cancer treatment, drug response prediction, and biomarker development [31, 32, 35]. [31, 32, 35–39] However, the random survival forest also has shortcomings: it is susceptible to outliers. Consequently, We combined two machine learning methods (LASSO algorithm and random survival forest) and traditional Cox regression to improve the accuracy of predictive models. Our multivariate survival analysis showed that SIRI and PLR had significant survival predictive values in patients with UTUC, consistent with previous studies. Therefore, we developed and constructed SIIS consisting of SIRI and PLR. High-risk patients had significantly worse overall survival (OS) than low-risk patients. A poor cancer prognosis is determined by elevated systemic inflammation responses (elevated neutrophil count and low lymphocyte count) [40]. The multivariate Cox regression model showed that high SIIS was considered an independent unfavorable prognostic indicator for OS in UTUC patients. In the past few years, many studies have begun to explore the correlation between inflammation and survival in cancer patients. However, those indicators are single indicators or generated by simple algorithms, which cannot fully reflect the immune and inflammatory state of patients, so the clinical application value is limited. For example, Zheng et al. incorporated SIRI and PLR into the models whose performance was higher than other indicators [18]. Jan et al. evaluated 424 patients with UTUC and demonstrated that the combination of high SII and high MLR has independent prognostic capacity in patients who underwent RNU. Although they have done much work, it is only a simple addition of these inflammatory-based factors. In this study, we compared the prognostic ability of SIIS and its components (NLR, MLR, PLR, SII, SIRI) for UTUC patients, and found that the predictive value of SIIS for patient survival was significantly higher than that of its components. Furthermore, a nomogram that combined SIIS and the other significant indicators indicated a high predictive performance of the model. In addition, the nomogram can distinguish patients into different groups with significant differences in OS.

The progression of tumor and inflammatory response is complex, with the fundamental processes underlying this response far from fully understood. The clinical significance of the SIIS might be explained by the neutrophils, platelets, monocytes, and lymphocytes. Immune and inflammatory cells (such as neutrophils, monocytes, and lymphocytes) are crucial components of the tumor microenvironment[9]. Therefore, neutrophils, monocyte, and lymphocytes might be necessary for cancer development and progression. Neutrophils are the first recruited effectors of the acute inflammatory response [41]. Neutrophils are modulated by tumor cells or other cells within the tumor microenvironment to infiltrate the tumor tissue and acquire tumor-promoting activities, such as angiogenesis, migration, invasion, metastasis, mutagenesis, or immunosuppression [42–45]. Moreover, A growing body of evidence supports a tumor-promoting role of a specific subpopulation of monocyte-derived macrophages, tumor-associated macrophages (TAMs), within the primary tumor microenvironment [10, 46]. TAMs play an essential role in tumor metastasis, involving almost every step of tumor cell metastasis, such as invasion, vascularization, establishing pre-metastatic niches, and so on[47]. Conversely, a relatively increasing number of circulating lymphocytes may reflect a higher level of cancer immune surveillance and defense [10, 48]. It is ascribed to the indispensable role of lymphocytes in cytotoxic cell death and cytokines secretion that suppress proliferation and metastatic activity of cancer cells [49]. The role of platelets in tumor progression has been recognized and reviewed in previous literature [50–53]. Folkman described that cancer progression with growth, tumorigenesis, and metastatic progression depends on abnormal angiogenesis for the first time[54]. An imbalance between proangiogenic factors and antiangiogenic factors regulates angiogenesis. These two factors are released from both tumor cells and platelets-derived microparticles. Platelet allows the tumor cell to progress and metastasis by involving in the different steps of angiogenesis(proliferation, migration, extracellular matrix degradation, and adhesion of endothelial cells) [55]. A new term: tumor-associated platelets—TAPs, has been introduced by Dymicka-Piekarska et al. [56]. Although there are no direct reports on the impact of blood platelets on tumor growth, tumor-associated platelets infiltrate the tumor environment in the same way that other cells do. Platelets, therefore, have the potential to influence the tumor microenvironment, induce neoangiogenesis and stimulate cancer progression [57]. Thus, an elevated SIIS resulted in a poor oncologic outcome.

However, there are several limitations to this work worth noting. It was a retrospective study with data collected from one center and lacked another independent external validation cohort. We should not ignore a certain selection bias inherent to any retrospective analysis. The current cutoff values chosen for serum markers resulted in an imbalanced grouping. Therefore, validating these conclusions through large-scale and high-quality prospective research in a multicenter is necessary. Second, the patients were treated by different doctors over a relatively long period, and the same pathologists did not confirm the specimens. So it was challenging to ensure the consistency of the clinicopathological data. Additionally, all patients included in our study were Chinese, so we cannot eliminate the influence of ethnic diversity.

## Conclusion

Our study applied the Lasso-Cox model to establish a novel systemic immune-inflammation score (SIIS). Our RSF model identified seven clinicopathological factors as important variables regarding overall survival. We combined the random survival forest model with the Cox proportional hazards regression model, and both models showed good predictive ability. Finally, Cox proportional hazards regression model was complementary to RSF models. We found that the preoperative elevated SIIS was associated with poor OS in the population of patients with UTUC who had undergone RNU. The nomogram model constructed by combining SIIS and other significant independent indicators had a good predictive performance. In addition, the nomogram had better discriminative power for patients with significantly different OS. The data suggest that the novel systemic immune-inflammation score could be a valuable biomarker for predicting outcomes.

## Data Availability

All data generated or analyzed during this study are included in this published article.

## List of abbreviations

AUROC: Area under the ROC curve
CI: Confidence interval
CIS: Carcinoma in situ
C-index: concordance index
CR: Complete response
CTU: Computed tomography
DCA: Decision curve analysis
DFS: Disease-free survival
LASSO: Least absolute shrinkage and selection operator
ROC: Receiver operating characteristic
HR: Hazard ratio
IVR: Intravesical recurrence (IVR)
LVI: Lympho-vascular invasion (LVI)
MLR: Monocyte-to-lymphocyte ratio (MLR)
MRI: Magnetic resonance imaging (MRI)
NLR: Neutrophil-to-lymphocyte ratio (NLR)
OOB: Out-of-bag
OS: Overall survival
PLR: Platelet-to-lymphocyte ratio
PUJ: Pelvi-ureteric junction
RNU: Radical nephroureterectomy
RSF: Random Survival Forest (RSF)
SII: Systemic immune-inflammation index (SII
SIIS: Systemic immune-inflammation score
SIRI: Systemic inflammation response index (SIRI)
TAMs: Tumor-associated macrophages (TAMs)
TAPs: Tumor-associated platelets—TAPs
Time-dependent AUC: Time-dependent receiver operating characteristic curve
UTUC: Upper urinary tract urothelial carcinoma
VIF: Variance inflation factor
VIMP: Variable importance
